# Principal component analysis identifies patterns of cytokine expression in non-small cell lung cancer patients undergoing definitive radiation therapy

**DOI:** 10.1371/journal.pone.0183239

**Published:** 2017-09-21

**Authors:** Susannah G. Ellsworth, Bryan M. Rabatic, Jie Chen, Jing Zhao, Jeffrey Campbell, Weili Wang, Wenhu Pi, Paul Stanton, Martha Matuszak, Shruti Jolly, Amy Miller, Feng-Ming Kong

**Affiliations:** 1 Department of Radiation Oncology, Indiana University School of Medicine, Indianapolis, Indianapolis, United States of America; 2 Department of Radiation Oncology, University of North Carolina, Chapel Hill, North Carolina, United States of America; 3 Department of Biostatistics & Epidemiology, Augusta University; Augusta, Georgia, United States of America; 4 Department of Radiation Oncology, Augusta University, Augusta, Georgia, United States of America; 5 Department of Radiation Oncology, University of Michigan School of Medicine, Ann Arbor, United States of America; University of South Alabama Mitchell Cancer Institute, UNITED STATES

## Abstract

**Background/Purpose:**

Radiation treatment (RT) stimulates the release of many immunohumoral factors, complicating the identification of clinically significant cytokine expression patterns. This study used principal component analysis (PCA) to analyze cytokines in non-small cell lung cancer (NSCLC) patients undergoing RT and explore differences in changes after hypofractionated stereotactic body radiation therapy (SBRT) and conventionally fractionated RT (CFRT) without or with chemotherapy.

**Methods:**

The dataset included 141 NSCLC patients treated on prospective clinical protocols; PCA was based on the 128 patients who had complete CK values at baseline and during treatment. Patients underwent SBRT (n = 16), CFRT (n = 18), or CFRT (n = 107) with concurrent chemotherapy (ChRT). Levels of 30 cytokines were measured from prospectively collected platelet-poor plasma samples at baseline, during RT, and after RT. PCA was used to study variations in cytokine levels in patients at each time point.

**Results:**

Median patient age was 66, and 22.7% of patients were female. PCA showed that sCD40l, fractalkine/C3, IP10, VEGF, IL-1a, IL-10, and GMCSF were responsible for most variability in baseline cytokine levels. During treatment, sCD40l, IP10, MIP-1b, fractalkine, IFN-r, and VEGF accounted for most changes in cytokine levels. In SBRT patients, the most important players were sCD40l, IP10, and MIP-1b, whereas fractalkine exhibited greater variability in CFRT alone patients. ChRT patients exhibited variability in IFN-γ and VEGF in addition to IP10, MIP-1b, and sCD40l.

**Conclusions:**

PCA can identify potentially significant patterns of cytokine expression after fractionated RT. Our PCA showed that inflammatory cytokines dominate post-treatment cytokine profiles, and the changes differ after SBRT versus CFRT, with vs without chemotherapy. Further studies are planned to validate these findings and determine the clinical significance of the cytokine profiles identified by PCA.

## Introduction

Although radiation therapy (RT) is primarily considered to be a local cancer treatment modality, it also induces a clinically significant systemic immune response. Circulating immunomodulatory factors known as cytokines are key modulators of the immune response to RT. It is thought that RT-induced cytokine release is associated both with systemic inflammation and augmentation of the immune response, as well as, paradoxically, with the development of acute and long-term immunosuppression. Presently, however, the cytokine response to RT is poorly understood. Gathering and analyzing data on cytokine expression in patients receiving RT is complicated by several factors. Cytokine expression exhibits diurnal variations [1] and changes in response to a variety of physiologic stressors including infection [2], cancer [3], and trauma [4]. The presence of large interindividual differences in cytokine responses further clouds the interpretation of cytokine expression patterns following radiation exposure [4]. Developing novel approaches to evaluating the complex cytokine response to RT is therefore critical in attempting to improve our understanding of the immunomodulatory effects of RT in cancer patients.

Prior analyses have identified certain (primarily inflammatory) individual cytokines as responsive to RT in patients with NSCLC [5, 6]. To date, however, a comprehensive analysis of cytokine expression patterns following RT has not been performed in lung cancer patients undergoing radiation. To address this deficit, we conducted a prospective study with the goal of collecting and analyzing a comprehensive panel of cytokines in NSCLC patients undergoing definitive RT and to attempt to correlate cytokine expression patterns during treatment with clinical outcomes. This study generated a complex dataset that includes levels of 30 different cytokines sampled from over 100 patients. This paper is the first report of this large dataset and will focus on changes in cytokine expression during RT.

Due to the complexity of the dataset, we used principal component analysis (PCA) to perform the data analysis. PCA, which transforms a large set of potentially inter-related variables into a smaller and ideally more manageable dataset of composite variables,[7] is well suited to performing exploratory analyses of complex data, and we selected this approach with the goal of identifying potentially significant patterns of cytokine expression changes in NSCLC patients undergoing radiation therapy while minimizing the risk of data loss.

## Methods

Patients undergoing definitive RT for NSCLC under IRB-approved institutional prospective protocols aimed at biomarker collection were eligible for this study, which enrolled 141 patients between March 2004 and April 2013. Written informed consent was obtained from each participant prior to study entry. The study was approved by the internal institutional review boards at each participating site (University of Michigan and Augusta University). All clinical investigations were conducted in accordance with the principles of the Declaration of Helsinki.

All patients were adults (at least 18 years of age); median age was 66 years. The majority of patients were men (77.3%), had a history of cigarette smoking, and had locally advanced disease. However, due to present standards of care for the treatment of non-small cell lung cancer, all patients in the SBRT group had earlier-stage (T1-T3, node negative) tumors. Further details of the patients’ clinical and demographic characteristics are presented in Table 1. Written informed consent was obtained from each participant prior to study entry. The first study was a dose escalation study using iso-toxicity criteria; the second was a dose escalation protocol using a PET-guided adaptive RT technique, with prescription dose limited by an iso-toxicity criterion, similar to that described in the RTOG 1106 study. The dose varied from 63–85.5 Gy in this protocol. In the second study, the radiation dose ranged from 66–90 Gy in 2.2- to 3.8-Gy fractions. The third study was an imaging and biomarker protocol using SBRT and CFRT with or without chemotherapy. The radiation dose was typically given as 60–74 Gy in 2-Gy fractions for CFRT or 50, 55, or 60 Gy in 10-, 11-, or 20-Gy fractions for SBRT.

**Table 1:** Basic clinical and demographic data.

	SBRT	ChRT	CFRT
**Age** (*years*; *median*, *range*)	73 (63–84)	64 (32–68)	73 (40–85)
**(n, %) Male**	10 (62.5%)	81 (75.7%)	16 (88.9%)
**(n, %) Female**	6 (37.5%)	26 (24.3%)	2 (11.1%)
**Tx**	0 (0%)	1 (1%)	0 (0%)
**T1**	7 (47.0%)	14 (13.1%)	6 (33.3%)
**T2**	6 (33.3%)	25 (23.4%)	6 (33.3%)
**T3**	3 (20.0%)	36 (32.7%)	3 (16.7%)
**T4**	0 (0%)	31 (29.0%)	3 (16.7%)
**N0**	16 (100%)	19 (17.8%)	11 (61.1%)
**N1**	0 (0%)	10 (9.3%)	2 (11.1%)
**N2**	0 (0%)	47 (43.9%)	3 (16.7%)
**N3**	0 (0%)	30 (28.0%)	2 (11.1%)
**Nx**	0 (0%)	1 (1%)	0 (0%)
**GTV size** (*cc; median*, *range*)	33.1(4.4–206.6)	165.6 (2.1–921.0)	187.7 (2.4–802.6)

Blood samples were collected at baseline and mid-treatment for SBRT, two weeks after starting CFRT. Samples were collected in K2EDTA-coated tubes for anticoagulation, placed on ice immediately, and centrifuged at 3,000g for 30 minutes. The supernatant was stored at -80°C until analysis. A commercially available human cytokine/chemoking magnetic bead panel kit (Milliplex^®^ MAP, Millipore, Billerica, MA, USA) was used to measure levels of the following cytokines: 1) EGF; 2) eotaxin; 3) fractalkine/CX3CL1; 4) G-CSF; 5) GM-CSF; 6) IFN-γ; 7) TGF-α; 8) TNF-α; 9) VEGF; 10) MCP-1; 11) IP-10/CXCL10; 12) MIP-1a; 13) MIP-1b; 14) sCD40l; 15) IL-1a; 16) IL-1b; 17) IL-1r; 18) IL-2; 19) IL-4; 20) IL-5; 21) IL-6; 22) IL-7; 23) IL-8; 24) IL-10; 25) IL-12p40; 26) IL-12p70; 27) IL-13; 28) IL-15; and 29) IL-17. TGF-ß1 levels were analyzed separately using a dedicated ELISA kit (Human TGF-ß1 Duoset kit, R&D Systems, Inc., Minneapolis, MN, USA). All samples were tested in duplicate. PCA was used to determine which cytokines explained the majority of the observed variations in cytokine levels due to RT. The Kruskal-Wallis test was used to compare median cytokine levels among the treatment groups.

## Results

### Study population

The present report includes results from 141 patients; PCA analysis was restricted to the 128 patients who had complete CK data at all prespecified time points of the analysis. Median age was 66, and 22.7% of patients were women. The majority of patients in all groups were white (124/128, 96%) and were either active smokers or had a history of cigarette smoking. Most patients had excellent performance status and no significant differences in baseline performance status were observed among the treatment groups (median baseline KPS in all three groups was 90). In keeping with established practice standards, all of the SBRT patients had N0 disease, in comparison to 61.1% of the CFRT patients (11/18) and 28.0% of the ChRT patients (30/107). Additionally, the treated volume was smaller in SBRT patients compared with the CFRT and ChRT patients (mean 33.1 cm^3^ in SBRT patients vs 165.6 cm^3^ and 187.7 cm^3^ in the CFRT and ChRt groups, respectively). Table 1 presents basic clinical and demographic data on the patients included in this report.

### PCA of cytokine levels in patients receiving RT

**Fig 1:**
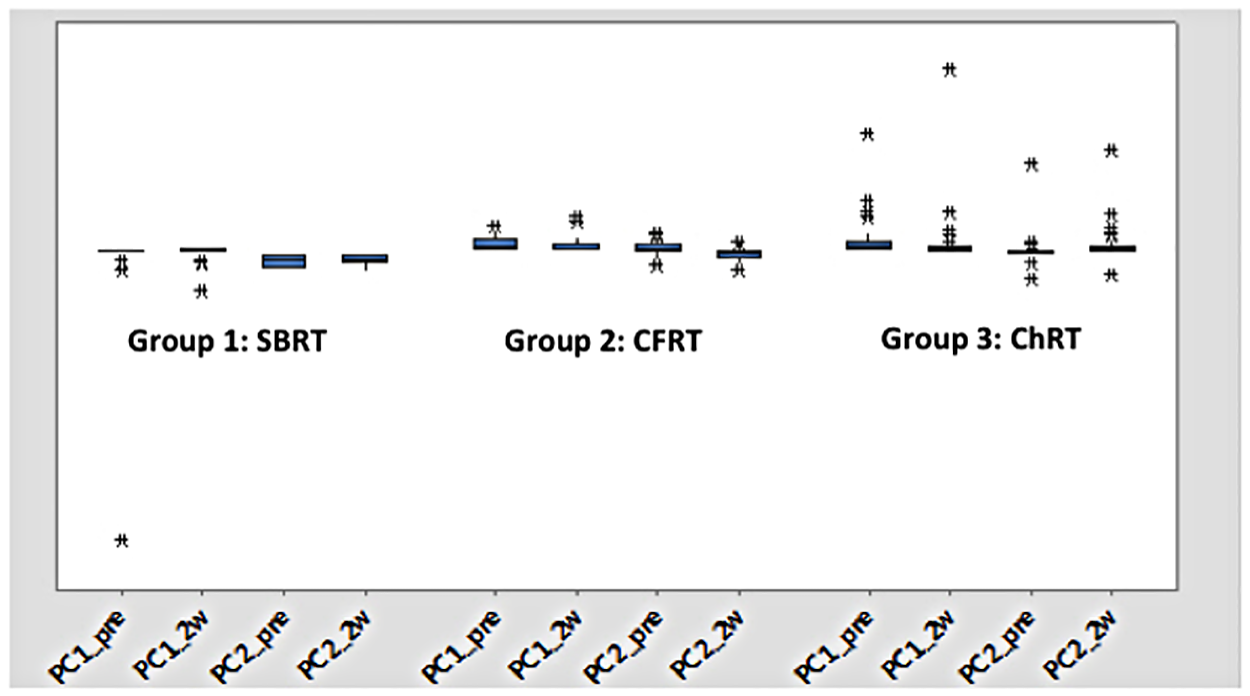
Graphical depiction of the PCA results at baseline and during RT. Group 1: SBRT; Group 2: CFRT; Group 3: ChRT. RT = Radiation therapy, SBRT = Stereotactic body radiation therapy (SBRT), CFRT = conventially fractionated radiation therapy, ChRT = Concurrent chemoradiation.

**Fig 2:**
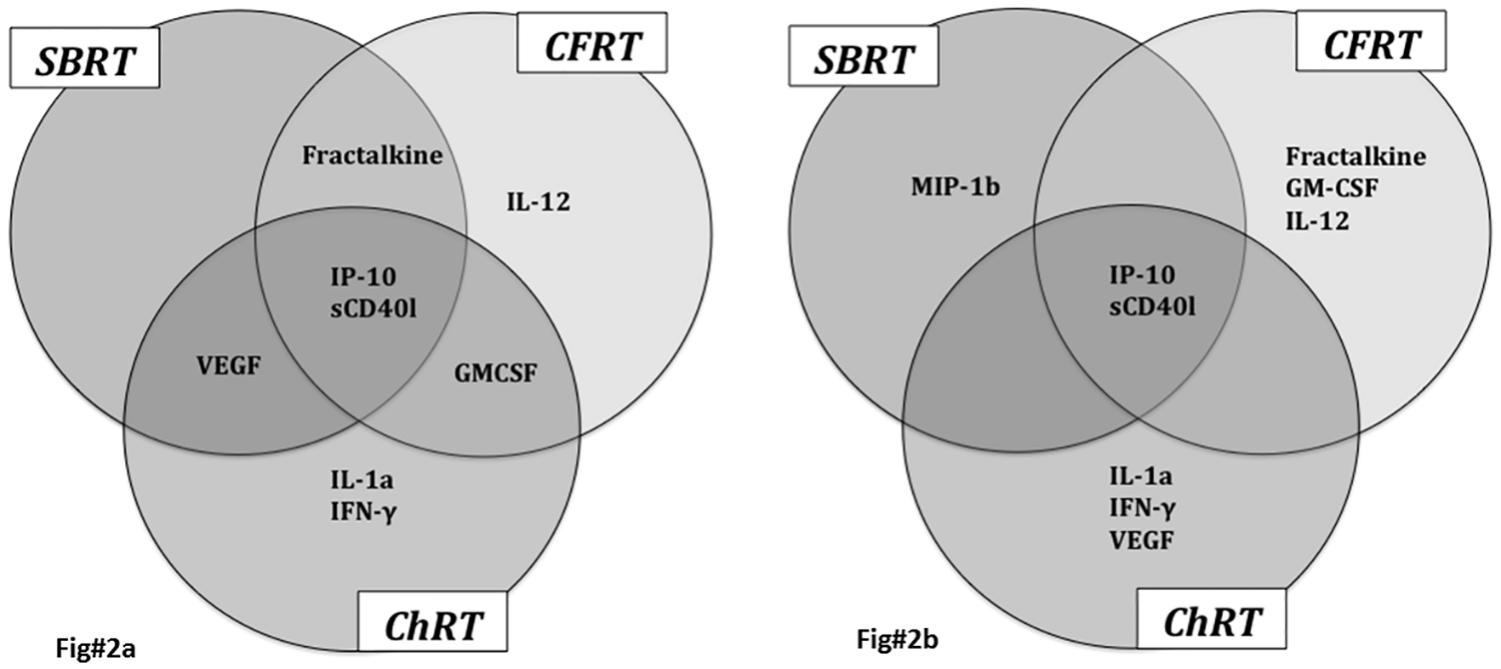
Venn diagrams showing cytokine expression patterns at baseline in the three treated groups at baseline (Fig 2a) and during RT (Fig 2b). SBRT = stereotactic body radiation therapy; CFRT = conventionally fractionated radiation therapy; ChRT = concurrent chemoradiation therapy. IL = interleukin; VEGF = vascular endothelial growth factor; GM-CSF = granulocyte-monocyte colony stimulating factor; IFN = interferon.

The following cytokines were identified by PCA as responsible for the bulk of the variations in cytokine levels at baseline and during RT: fractalkine/CX3CL1, GM-CSF, IL-1a, IL-12 (p40), IFN-γ, IP-10, MIP-1b, sCD40l, and VEGF. Fig 1 graphically shows the leading PCs identified in our analysis. As shown in the figure, SBRT patients expressed a more limited repertoire of cytokines and less variability in cytokine levels, both at baseline and during treatment, than either the CFRT or ChRT patients. IP10 and sCD40l were identified as significant players in all three groups both at baseline and during treatment. Otherwise, cytokine expression patterns varied among the treatment groups, as shown in Fig 2a and 2b. Fractalkine/CX3CL1 appeared to influence cytokine levels in SBRT and CFRT patients but not in the ChRT group, whereas VEGF was identified as a potentially significant cytokine in SBRT and ChRT patients but not CFRT patients. GM-CSF was also involved in cytokine expression patterns at baseline in non-SBRT patients (i.e., CFRT and ChRT patients), whereas IL-12 was only identified as a potentially significant factor at baseline in CFRT patients. The ChRT group appeared to be more influenced by IFN-γ and IL-1a, whereas these cytokines did not appear to play as important a role in the RT-alone groups (i.e., SBRT and CFRT patients).

PCA also identified specific cytokines as potentially major players in the response to RT. For patients treated with SBRT, IP-10, MIP-1b, and sCD40l were responsible for the majority of the variation in cytokine levels seen with treatment. In patients undergoing CFRT, fractalkine/CX3CL1, IP-10, IL-12, GMCSF, and sCD40l appeared to be the most important cytokines; and in ChRT patients, IP-10, MIP-1b, and sCD40l accounted for the majority of the variability in cytokine levels during treatment, in addition to IFN-γ, IL-1a and VEGF. Interestingly, IL-1a and IFN-γ were again identified as major players only in the ChRT patients. The reported PCs accounted for 99.5% and 94.8% of the variability observed at baseline and during treatment, respectively. We also analyzed changes in levels of the cytokines identified by PCA as potentially key players among the three different treatment groups over time; these data are shown in Table 2.

**Table 2:** Median cytokine values at baseline and during treatment for cytokines identified as key players by PCA.

*FRACTALKINE*				
	**CFRT**	**ChRT**	**SBRT**	***P***
**Baseline (pre)**	92.19	86.07	69.47	0.83
**During (w2)**	62.83	76.77	66.25	0.81
**Δ (%)**	-31.8	-10.8	-4.6	
*GM-CSF*				
	**CFRT**	**ChRT**	**SBRT**	
**Baseline (pre)**	13.74	21.04	9.70	0.03
**During (w2)**	14.31	16.03	13.15	0.24
**Δ (%)**	+4.1	-23.8	+35.6	
*IFN-γ*				
	**CFRT**	**ChRT**	**SBRT**	
**Baseline (pre)**	10.95	10.24	7.56	0.24
**During (w2)**	29.05	8.47	7.42	0.25
**Δ (%)**	+165.2	-17.3	-1.9	
*IL-12p40*				
	**CFRT**	**ChRT**	**SBRT**	
**Baseline (pre)**	105.95	3.55	3.20	0.08
**During (w2)**	84.42	3.20	3.20	0.03
**Δ (%)**	-20.3	-9.9	0	
*IL-1a*				
	**CFRT**	**ChRT**	**SBRT**	
**Baseline (pre)**	33.11	16.91	9.03	0.31
**During (w2)**	27.39	12.11	9.96	0.62
**Δ (%)**	-17.3	-28.4	+10.3	
*IP-10*				
	**CFRT**	**ChRT**	**SBRT**	
**Baseline (pre)**	525.06	524.95	591.39	0.40
**During (w2)**	513.25	409/84	567.54	0.36
**Δ (%)**	-2.2	-21.9	-4.0	
*MIP-1b*				
	**CFRT**	**ChRT**	**SBRT**	
**Baseline (pre)**	40.72	45.70	33.77	0.83
**During (w2)**	47.04	43.79	36.89	0.95
**Δ (%)**	+15.5	-4.2	+9.2	
*sCD40l*				
	**CFRT**	**ChRT**	**SBRT**	
**Baseline (pre)**	380.08	493.08	277.75	0.15
**During (w2)**	368.58	406.26	300.09	0.37
**Δ (%)**	-3.0	-17.6	+8.0	
*VEGF*				
	**CFRT**	**ChRT**	**SBRT**	
**Baseline (pre)**	82.57	78.06	71.78	0.97
**During (w2)**	67.21	69.91	64.26	0.89
**Δ (%)**	-18.6	-10.4	-10.5	

## Discussion

The present study is the largest to date to prospectively collect and analyze a comprehensive panel of cytokines in NSCLC patients undergoing definitive RT and to attempt to correlate cytokine expression patterns with clinical outcomes. Our analysis demonstrates that PCA is a potentially powerful tool for analyzing the complex patterns of cytokine release following RT in patients with lung cancer and may provide additional information regarding potentially clinically significant cytokines compared to an analysis of changes in median or mean levels alone. PCA is a well-described statistical tool that can be used to analyze complex multidimensional datasets and is frequently employed to identify hypothesis-generating patterns of interest in the context of exploratory analyses such as the present study. [8] PCA is well suited to the analysis of patterns of cytokine expression following radiation therapy, which is complicated by several interrelated factors: 1) interindividual variability in RT-induced cytokine release; 2) the release of certain significant cytokines, such as TGF-β1, by tumors themselves; 3) variations in physical factors, such as radiation dose and the irradiated volume, that could influence the timing and magnitude of cytokine release; and 4) the complex regulatory interplay among clinically significant cytokines that are released following RT. An additional advantage of the use of PCA in the present study, which tested levels of 30 different cytokines at multiple time points, is that this technique can reduce confounding due to multiple hypothesis testing, a common pitfall when analyzing large and complicated datasets such as this.

Our work has identified fractalkine/CX3CL1, GM-CSF, IL-1a, IL-12 (p40), IFN-γ, IP-10, MIP-1b, sCD40l, and VEGF as cytokines of possible clinical importance in NSCLC patients who undergo RT. We also observed significant variability in cytokine expression patterns among the different treatment groups both before and during RT. Interestingly, neither the direction nor the magnitude of change in median cytokine level was consistently associated with whether or not a particular cytokine had been identified by PCA as potentially significant, suggesting that PCA may provide additional information beyond descriptive statistics (e.g., median and/or mean) in identifying cytokines of potential clinical importance.

The observed variations before RT may have been due to heterogeneity in baseline characteristics of the patient population, such as age, differences in the tumor burden, or medical comorbidities. Changes during RT may have resulted from a combination of tumor and normal tissue responses to treatment. SBRT patients demonstrated the least amount of variability in cytokine expression at baseline and during RT, perhaps due to the fact that SBRT patients had earlier-stage disease and underwent more conformal treatments. Additionally, these findings may reflect differences in the inflammatory/immune response to the high-dose, short-course RT administered to SBRT patients or differences in the timing of plasma collection relative to RT administration.

During RT, PCA showed that sCD40l and IP-10 were major players in all three treatment groups, reflecting a primarily inflammatory picture which is consistent with previously described effects of irradiation on the lung parenchyma [6, 9]. Soluble CD40 ligand (sCD40l) is an 18-kDa protein that is primarily released by activated T cells and platelets. [10] It has been primarily studied within the context of autoimmune diseases and cardiovascular pathology; however, there is some evidence that it plays a role in malignant disease as well. [11] For example, levels of this protein may be correlated with disease burden in patients with small cell lung cancer and nasopharyngeal carcinoma. [12, 13] The CD40 receptor itself is involved in a variety of immune and inflammatory responses, and ligand binding is associated with the release of IL-6, IL-10, and IL-12. Interestingly, increased sCD40l levels may be associated with an immunosuppressed phenotype, as ligand binding has been shown to stimulate both myeloid and lymphoid suppressor cells as well as PD-1 expression in cancer patients. [14]

IP-10 (also known as CXCL10) and MIP-1β (also referred to as CCL4) are well established pro-inflammatory cytokines. IP-10 has been shown to play a role in several autoimmune diseases and is associated with the stimulation of an antiviral immune response in hepatitis C infection. [15] Early increases in IP-10 have been associated with the risk of higher-grade pulmonary toxicity in lung cancer patients treated with radiation therapy. [5] Limited clinical data are available regarding the effects of MIP-1β in irradiated individuals, but it also appears to be an immunostimulatory chemokine that directs leukocyte migration following tissue injury. [16]

Fractalkine (or CX3CL1) is the only member of a unique subclass of chemokines that exists in both a soluble and a membrane-bound form. Like the other cytokines consistently identified as potentially important factors in our analysis, it is a pro-inflammatory molecule that is clinically associated with vascular pathology as well as autoimmune diseases including rheumatoid arthritis. Its role in malignancy is controversial, with some studies appearing to show that fractalkine has antitumor activity, and other investigations suggesting that fractalkine may play a pro-metastatic role in some settings. [17] Fractalkine levels have also been shown to correlate with tumor burden in NSCLC. [18] It is interesting to note that this cytokine was identified as a major player by PCA at baseline in the SBRT and CFRT groups, but lost significance during treatment in the SBRT patients, which may be suggestive of an early response to ablative dose radiation therapy.

Additional cytokines of interest identified by the PCA include VEGF, GM-CSF, IL-12, IFN-γ, and IL-1a. Although a detailed description of the potential role of each of these individual substances in lung cancer is beyond the scope of this paper, each of these cytokines has been identified in the past as either an inflammatory (VEGF, GM-CSF, IL-12, IFN-γ, IL-1a) or a potentially antitumor (IL-12, IFNγ) cytokine. IL-8 and TGF-β have additionally been identified by prior studies as clinically significant cytokines in NSCLC patients treated with radiation. [9] Elevated TGF-β levels have been associated with a higher risk of radiation pneumonitis in several published series, as have low levels of IL-8. [19] It is unclear why neither IL-8 nor TGF-β was identified by PCA as a potentially important player in the present analysis, but one contributing factor may be the relatively low rate of grade 2 or greater pneumonitis (approximately 20%) observed in our dataset, allowing tumor-related, rather than-toxicity-related, effects to predominate.

Limitations of the present work include the fact that PCA can only be used to identify potentially important contributors to the observed patterns of cytokine expression. PCA is unable to provide detailed information regarding quantitative correlations between cytokine levels and biologic/clinical outcomes of interest. Additionally, the number of SBRT patients included in the dataset is relatively small, limiting our ability to draw definitive conclusions from the data at hand. Furthermore, we are awaiting the results of additional analyses that correlate levels of potentially clinically significant individual cytokines identified by PCA with clinical outcomes including tumor control, patterns of failure, survival, and the development of radiation-induced toxicity. Nevertheless, we believe that our analysis demonstrates the utility of PCA as a statistical tool for parsing large and complex datasets such as the comprehensive panel of cytokine expression analyzed herein and identifying candidates for more detailed future analyses.

In conclusion, our analysis has demonstrated that PCA is a powerful statistical tool for analyzing cytokine expression profiles in patients undergoing RT. Despite the finding of a primarily inflammatory cytokine profile in all patients during RT, cytokine expression patterns in these patients vary significantly by treatment group, likely reflecting differences in tumor burden as well as variations in the immune system’s response to alterations in target volume size, daily radiation dose, and the use of chemotherapy. Based on the results of the PCA, further studies are planned to determine whether cytokine expression patterns in NSCLC patients, and specifically levels of the major players identified by PCA, are associated with other clinical outcomes including high-grade toxicity, patterns of failure, and tumor control.

## Supporting information

S1 Dataset Complete clinical cytokine dataset. Anonymized clinical database on which this report is based.
(PDF)
