# Supplementary material for: Principal component analysis identifies patterns of cytokine expression in non-small cell lung cancer patients undergoing definitive radiation therapy
Source: PLoS One. 2017 Sep 21;12(9):e0183239. doi: 10.1371/journal.pone.0183239 (PMC5608186; doi:10.1371/journal.pone.0183239)
Supplement: S1 Dataset — Anonymized clinical database on which this report is based. (PDF) [file pone.0183239.s001.pdf]

Unique No.	Gender	Race	Tx Group	RT Dose	Dose per fx	BED10	EQD2	KPS
1	M	w	CFRT	60.00	2.0*30	72.00	60.00	90
12	M	w	CFRT	70.00	2.0*35	84.00	70.00	90
21	M	w	CFRT	70.00	2.0*35	84.00	70.00	70
22	M	w	CFRT	75.60	2.1*36	91.48	76.23	90
23	F	w	CFRT	66.00	2.0*33	79.20	66.00	90
24	M	w	CFRT	75.60	2.1*36	91.48	76.23	90
26	M	w	CFRT	64.00	2.0*32	76.80	64.00	80
41	M	w	CFRT	70.00	2.0*35	84.00	70.00	70
59	M	w	CFRT	66.00	2.0*33	79.20	66.00	80
67	M	w	CFRT	60.00	2.0*30	72.00	60.00	70
72	M	w	CFRT	64.00	2.0*32	76.80	64.00	90
87	M	w	CFRT	60.00	2.0*30	72.00	60.00	100
88	M	w	CFRT	74.40	1.8*25+1.85*16	93.62	78.02	90
91	M	w	CFRT	70.00	2.0*35	84.00	70.00	100
97	M	w	CFRT	34.00	2.0*17	40.80	34.00	70
113	M	w	CFRT	85.50	2.85*12+2.85*18	109.87	91.56	80
145	F	w	CFRT	87.90	2.1*24+2.5*15	107.86	89.88	70
183	M	w	CFRT	60.00	2.0*30	72.00	60.00	90
2	M	w	CHRT	67.80	2.0*18+2.65*12	83.43	69.52	90
3	M	w	CHRT	73.00	2.0*18+3.15*12	91.50	76.25	90
4	M	w	CHRT	64.20	2.0*18+1.8*16	78.03	65.02	90
5	M	w	CHRT	64.20	2.0*18+2.35*12	78.03	65.02	90
6	M	w	CHRT	63.80	2.0, 2.55 and 2.3	78.03	65.03	100
7	M	w	CHRT	63.00	2.1*30	76.23	63.53	90
8	M	w	CHRT	75.60	2.1*36	91.48	76.23	90
10	M	w	CHRT	45.00	2.5*18	56.25	46.88	70
15	F	w	CHRT	63.00	2.1*30	76.23	63.53	90
16	M	w	CHRT	66.00	2.0*33	76.80	64.00	65
17	M	w	CHRT	81.40	2.19*8+2.0*10+3.57*	103.24	86.03	100
18	M	w	CHRT	65.40	2*3*6+2.45*2*6	78.81	65.67	90
19	M	w	CHRT	64.20	2.0*18+2.35*2*6	78.03	65.02	85
20	M	w	CHRT	64.20	2.0*18+2.35*2*6	78.03	65.02	80
25	F	w	CHRT	73.20	2.0*6*6+3.1*2*6	91.93	76.61	80
27	M	w	CHRT	64.00	2.0*18+2.35*2*6	76.80	64.00	90
28	M	w	CHRT	64.20	2.0*18+2.35*2*6	78.03	65.02	90
30	M	w	CHRT	68.20	2.0*20+2.35*2*6	83.04	69.20	90
32	M	w	CHRT	64.20	2*18+2.35*2*6	78.03	65.02	95
33	M	w	CHRT	66.00	2.0*33	79.20	66.00	80
34	F	w	CHRT	66.00	2.0*33	79.20	66.00	90
36	F	w	CHRT	70.00	2.0*35	84.00	70.00	50

38	M	w	CHRT	70.00	2.0*35	84.00	70.00	90
39	M	w	CHRT	70.00	2.0*35	84.00	70.00	90
40	F	w	CHRT	66.00	2.0*33	79.20	66.00	90
42	M	w	CHRT	60.00	2.0*30	72.00	60.00	90
44	M	w	CHRT	70.00	2.0*35	84.00	70.00	90
45	M	w	CHRT	70.00	2.0*35	84.00	70.00	90
46	M	w	CHRT	66.00	2.0*33	79.20	66.00	90
47	M	w	CHRT	44.00	2.0*22	52.80	44.00	90
48	M	w	CHRT	70.00	2.0*35	84.00	70.00	90
49	M	w	CHRT	70.00	2.0*35	84.00	70.00	90
51	M	w	CHRT	70.00	2.0*35	84.00	70.00	90
53	M	w	CHRT	70.00	2.0*35	84.00	70.00	90
54	M	w	CHRT	72.00	2.4*30	86.40	72.00	60
55	M	w	CHRT	74.00	2.0*37	88.80	74.00	80
56	M	w	CHRT	74.00	2.0*37	88.80	74.00	80
57	M	w	CHRT	66.00	2.0*33	79.20	66.00	80
58	M	w	CHRT	74.40	2.4*31	93.62	78.02	80
60	M	w	CHRT	60.00	2.0*30	72.00	60.00	80
62	F	w	CHRT	70.00	2.0*35	84.00	70.00	80
63	M	w	CHRT	74.40	2.4*31	93.62	78.02	80
64	M	w	CHRT	60.00	2.0*30	72.00	60.00	90
65	M	w	CHRT	52.00	2.0*26	62.40	52.00	90
66	F	w	CHRT	69.30	2.1*24+3.15*6	85.81	71.51	90
68	M	w	CHRT	62.00	2.0*31	74.40	62.00	80
73	M	w	CHRT	66.00	2.0*33	79.20	66.00	70
74	M	w	CHRT	85.50	2.85*30	109.87	91.56	90
77	M	w	CHRT	70.00	2.0*35	84.00	70.00	90
80	F	w	CHRT	66.00	2.0*33	79.20	66.00	60
81	M	w	CHRT	74.00	2.0*37	88.80	74.00	80
83	F	w	CHRT	85.50	2.85*30	109.87	91.56	70
84	F	w	CHRT	85.40	2.7*19+3.1*11	109.82	91.52	90
86	M	w	CHRT	66.00	2.0*33	79.20	66.00	70
89	M	w	CHRT	60.00	2.0*30	72.00	60.00	80
90	F	w	CHRT	85.50	2.85*30	109.87	91.56	70
92	M	w	CHRT	74.00	2.0*37	88.80	74.00	85
93	M	w	CHRT	74.00	2.0*37	88.80	74.00	80
94	F	w	CHRT	78.35	2.15*13+2.4*9+3.6*8	100.49	83.74	100
95	M	w	CHRT	82.65	2.35*21+3.7*9	106.57	88.81	90
96	M	w	CHRT	70.00	2.0*35	84.00	70.00	80
98	F	w	CHRT	85.15	2.6*19+3.25*11	109.61	91.34	85
101	M	w	CHRT	85.50	2.85*30	109.87	91.56	80
102	M	w	CHRT	71.60	2.2*23+3.0*7	89.03	74.19	90

103	M	w	CHRT	85.50	2.85*30	109.23	91.03	80
104	M	w	CHRT	85.50	2.85*30	109.87	91.56	100
106	M	w	CHRT	85.55	2.65*19+3.2*11	110.16	91.80	100
107	M	w	CHRT	74.40	2.2*23+3.4*7	93.62	78.02	90
108	M	w	CHRT	70.45	2.15*23+3.0*7	87.38	72.82	80
109	M	w	CHRT	76.00	2.5*20+2.6*10	95.26	79.38	90
110	M	w	CHRT	73.50	2.25*22+3.0*8	91.84	76.53	80
111	M	w	CHRT	70.00	3.5*20	84.00	70.00	90
112	M	w	CHRT	66.00	2.0*33	79.20	66.00	90
114	M	w	CHRT	85.40	2.7*19+3.1*11	109.82	91.52	80
115	M	w	CHRT	84.50	2.4*20+3.65*10	109.34	91.12	80
116	M	w	CHRT	85.25	2.6*19+3.25*11	109.61	91.34	100
118	M	w	CHRT	85.50	2.85*30	109.87	91.56	80
119	F	w	CHRT	63.00	2.1*30	76.23	63.53	100
120	F	w	CHRT	85.00	2.5*20+3.5*10	109.75	91.46	100
121	M	w	CHRT	70.00	2.15*23+2.95*7	86.82	72.35	70
122	M	w	CHRT	85.50	2.85*30	109.87	91.56	70
123	M	w	CHRT	77.20	2.2*23+3.8*7	98.44	82.03	80
124	M	w	CHRT	83.50	2.45*20+3.45*10	107.41	89.51	80
125	M	w	CHRT	78.50	2.45*20+2.95*10	99.21	82.68	85
127	F	w	CHRT	85.00	2.5*20+3.5*10	109.75	91.46	90
128	M	w	CHRT	85.50	2.85*30	109.87	91.56	80
130	M	w	CHRT	78.00	2.55*20+2.7*10	98.30	81.92	90
132	M	w	CHRT	73.50	2.45*30	91.51	76.26	100
134	F	w	CHRT	77.20	2.2*23+3.8*6	93.20	77.67	90
135	F	w	CHRT	81.50	2.5*20+2.15*14	99.07	82.56	90
136	F	w	CHRT	66.00	2.0*33	79.20	66.00	90
139	M	w	CHRT	55.00	2.0*27+2.2*4	115.50	96.25	90
142	M	w	CHRT	70.00	2.0*35	84.00	70.00	85
143	F	w	CHRT	60.00	2.0*30	72.00	60.00	80
146	F	w	CHRT	78.00	2.45*20+2.9*10	98.42	82.01	85
147	M	w	CHRT	74.10	2.35*21+2.75*9	92.50	77.09	90
148	M	w	CHRT	82.10	2.2*23+4.5*7	107.41	89.51	100
149	F	w	CHRT	85.50	2.85*30	109.87	91.56	90
150	M	w	CHRT	60.00	2.0*30	72.00	60.00	90
152	F	w	CHRT	66.00	2.0*33	79.20	66.00	90
153	M	w	CHRT	77.25	2.35*21+3.1*9	97.50	81.25	90
154	M	w	CHRT	64.00	2.0*32	76.80	64.00	70
155	M	w	CHRT	66.00	2.0*33	79.20	66.00	90
161	M	w	CHRT	60.00	2.0*30	72.00	60.00	80
162	M	b	CHRT	60.00	2.0*30	72.00	60.00	90
164	M	w	CHRT	74.00	2.0*37	88.80	74.00	90

167	F	w	CHRT	60.00	2.0*30	79.20	66.00	100
169	F	w	CHRT	60.00	2.0*30	72.00	60.00	100
170	M	b	CHRT	70.00	48.4 in 2.8 and 21.6 ir	89.38	74.49	100
171	M	w	CHRT	66.00	2.0*33	79.20	66.00	80
176	F	w	CHRT	60.00	2.0*30	72.00	60.00	90
177	F	asian	CHRT	60.00	2.0*30	72.00	60.00	100
178	F	w	CHRT	60.00	2.0*30	72.00	60.00	90
189	M	w	CHRT	66.00	2.0*30	79.20	66.00	80
69	F	b	SBRT	55.00	5.0*11	115.50	96.25	100
70	M	w	SBRT	55.00	5.0*11	115.50	96.25	90
71	F	w	SBRT	60.00	20.0*3	180.00	150.00	90
82	F	w	SBRT	50.00	5.0*10	100.00	83.33	50
105	M	w	SBRT	54.00	11*3+5*2+11*1	151.20	126.00	90
129	M	w	SBRT	55.00	5.0*11	115.50	96.25	70
131	M	w	SBRT	55.00	5.0*11	115.50	96.25	85
133	M	w	SBRT	55.00	5.0*11	115.50	96.25	80
141	M	w	SBRT	55.00	5.0*11	115.50	96.25	100
144	M	w	SBRT	50.00	10.0*5	100.00	83.33	90
157	F	b	SBRT	55.00	5.0*11	115.50	96.25	70
160	M	w	SBRT	55.00	5.0*11	115.50	96.25	90
163	F	w	SBRT	55.00	5.0*11	115.50	96.25	60
165	F	w	SBRT	55.00	5.0*11	115.50	96.25	90
173	M	w	SBRT	50.00	5.0*10	100.00	83.33	70
188	M	w	SBRT	55.00	11.0*5	115.50	96.25	70
175	M	w		45.00	1.5*30	51.75	43.13	100
179	F	w		60.00	2.0*30	72.00	60.00	70
185	M	w		62.00	2.0*31	74.40	62.00	90
187	M	w		66.00	2.0*33	79.20	66.00	90

SimpleStage	T Stage	N stage	M stage	GTV volume (cc)	pre_il2	pre_ip10	pre_scd40l	pre_TGFbeta1
1	1	0	0	15.20	66.28	100.01	1264.92	14.12
1	1.5	0	0	12.63	47.39	142.32	1060.90	15.74
1	2	0	0	185.07	1.06	975.42	315.92	4.21
1	1	0	0	2.07	15.84	350.99	274.85	23.74
1	1	0	0	25.96	11.25	162.69	164.36	14.59
1	1	0	0	12.04	12.55	707.41	214.77	3.73
2	2	0	0	197.59	14.52	752.82	366.00	12.08
2	3	0	0	150.38	22.43	815.44	1197.11	16.35
3	2	2	0	79.88	2.00	341.16	345.12	5.00
3	4	2	0	288.01	1.42	694.65	818.02	5.84
3	4	1	0	445.58	6.56	2329.79	658.84	2.56
3	1	2	0	91.63	3.91	709.89	380.08	1.35
3.5	4	0	0	158.89	0.2	3.2		5.1368715
1	1	0	0	47.60	0.12	3.2		5.4494185
2.5	3	1	0	921.00	0.345	3.2		14.660792
2	3	0	0	9.79	4.38	355.465	905.54	5.6354857
3.5	2	3	0	171.70	14.925	808.885	2787.14	7.6615818
3	2	3	0		8.34	1874.045	210.575	
3	4	2	0	582.01	2.00	292.47	485.94	5.24
3.5	4	1	0	164.68	33.69	457.31	331.62	3.05
3	4	0	0	491.77	16.60	381.75	213.92	10.42
3.5	3	3	0	223.72	5.94	824.02	27.54	3.36
3	X	2	0	297.37	110.63	901.34	1297.53	5.40
2	3	0	0	98.55	143.44	632.68	3592.87	36.08
2	3	0	0	81.56	66.02	222.02	1048.90	13.72
3	3	3	0	164.03	29.98	155.74	702.81	26.02
3.5	3	3	0	643.98	10.88	827.63	228.45	8.14
3	3	1	0	628.70	6.27	432.65	345.23	10.52
3.5	4	2	0	81.52	17.11	885.19	181.15	35.00
3.5	3	3	0	541.96	9.70	365.26	496.10	23.94
3	2	2	0	367.99	11.19	374.50	485.31	4.92
3	3	3	0	802.85	21.50	229.92	493.08	27.85
3	3	2	0	52.57	48.42	262.16	703.98	15.59
3.5	4	2	0	133.02	2.00	747.09	1431.00	2.64
3	3	2	0	217.98	1.90	317.56	1564.12	3.02
3	2	2	0	266.64	52.67	173.12	1613.71	14.39
3	1	2	0	66.56	2.00	60.11	447.62	2.74
3	1	2	0	83.48	12.04	838.38	408.48	5.50
3	3	2	0	235.26	3.07	340.51	202.43	12.78
3.5	4	3	0		8.33	530.16	334.43	2.48

3	4	2	0	446.37	180.57	567.36	262.36	1.92
1	1	0	0	2.35	1.39	259.24	314.76	11.43
3	1	2	0	37.65	1.63	193.65	731.95	14.92
3	2	2	0	78.10	0.57	175.11	170.22	6.87
3	1	2	0	118.11	1.06	258.51	186.03	2.60
3	3	2	0	222.72	4.97	491.65	239.64	10.69
3	4	3	0	337.59	0.04	299.33	75.51	8.62
3	3	2	0		6.05	1062.19	235.08	2.76
3	4	2	0	109.57	20.08	134.11	334.68	4.32
3	1	2	0	21.76	23.22	261.27	305.75	3.42
3	4	2	0	302.29	0.87	459.05	411.04	5.68
3	4	1	0	251.24	2.00	348.84	344.91	3.64
1	1	0	0	8.02	2.00	909.46	373.10	3.20
3	3	2	0	55.83	74.59	429.62	1183.38	7.10
2	3	0	0	94.77	88.66	682.50	619.77	2.20
3	3	3	0	100.12	274.69	1493.42	1046.76	5.00
1	2	0	0	14.34	2.00	1099.79	255.66	3.20
3	3	3	0	101.33	2.00	987.09	1088.57	4.12
3	4	2	0	275.97	2.00	612.70	815.35	9.55
1	2	0	0	56.23	2.00	916.36	812.54	1.32
3	2	2	0	164.43	2.00	693.36	457.05	1.80
3	2	2	0	123.03	516.44	1626.81	10000.00	1.33
3.5	3	3	0	393.94	12.15	533.62	1060.02	15.97
1	2	0	0	249.23	10.07	576.06	1435.25	20.12
3	2	3	0	324.70	0.99	752.06	819.33	2.82
3	3	3	0	433.31	27.97	979.80	1256.82	21.07
3	4	1	0	88.96	20.91	492.50	496.87	1.09
3	4	3	0	157.00	4.22	661.42	131.18	1.73
3	1	3	0	43.02	3.94	745.75	148.93	2.05
3	4	0	0	93.96	4.42	80.32	94.48	5.57
1	2	0	0	58.00	4.36	1149.60	126.65	4.10
3.5	4	0	0	251.75	4.93	397.44	381.33	4.40
3.5	3	3	0	138.59	0.7	3.2		13.285001
3.5	4	3	0	225.19	0.775	3.2		7.171348
3.5	4	2	0	272.14	0.495	3.2		7.4985
3.5	4	2	0	392.48	312.2175	402.9987	0	
3.5	4	2	0	184.77	0.275	3.2		3.382811
3	4	1	0	272.22	0.775	3.2		6.5681615
3	2	2	0	319.43	0.12	3.2		4.6052495
3	3	1	0	19.19	4.46	22.68		11.053357
3	1	2	0	26.35	0.345	3.2		4.4212265
3.5	2	3	0	117.46	4.97	3.2		10.425342

3.5	1	3	0	81.66	21.85	939.99	623.34	5.8567006
3.5	4	0	0	62.89	15.33	524.95	538.675	6.0747777
3.5	2	3	0	162.12	0.64	538.53	238.895	1.7822673
3	3	2	0	250.00	3.36	978.69	1707.13	8.1425879
3	1	2.0	0.0	75.73	0.64	730.51	387.37	0.8519096
3.5	4	0	0	206.61	11.38	689.17	1802.5	4.3474188
3.5	4	2	0	186.33	14	421	566.05	7.1322163
1	2	0	0	21.56	4.545	379.29	568.545	3.3166515
3	3	3	0	42.30	1.27	500.325	879	3.1880017
3.5	4	1	0	126.25	11.08	229.735	323.02	
3.5	3	3	0	103.23	9.425	1360.72	411.345	3.1723127
3.5	4	2	0	299.64	3.48	2198.32		4.8948864
2.5	2	1	0	32.70	23.90	356.36		20.7584024
3.5	1	3	0	19.00	4.55	2184.06		4.8074776
3.5	4	2	0	57.98	9.22	1294.96		7.17224
3	3	2	0	616.81	0.00	699.65		5.731176
2.5	3	0	0	112.05	3.48	1228.60		3.8790544
3.5	4	0	0	359.11	9.23	804.51		11.9513752
3.5	4	2	0	361.13	1.20	1350.54		4.4885536
3.5	3	3	0	176.17	0.32	1708.34		3.9877248
3	2	2	0	102.02	9.15	912.37		6.3028768
2.5	3	0	0	70.64	0.00	2098.49		4.4507552
3	2	2	0	180.09	3.885	197.13	1045.725	9.7141936
3	3	2	0	80.42	5.02	1489.515	660.44	4.7873606
3.5	3	3	0	113.42	6.5	199.475	3420.8	8.3589616
3.5	3	3	0	76.70	0.39	1035.115	340.83	2.545581
3	1	2	0	18.60	176.35	1076	1463.035	3.011442
3.5	3.5	3	0	165.00	8.31	485.17	931.62	
2.5	2	1	0	34.70	0.62	234.135	807.745	5.3859214
3.5	3	3	0	20.63	2.825	278.335	796.14	6.103065
3	3	2	0	154.50	3.12	661.28	874.54	
3	1.5	2	0	98.80	4.66	225.94	22.495	
3	3	2	0	45.00	1.9	371.105	0.4	
3	1	2	0	69.42	3.555	534.185	0.4	
3	2	2	0	466.91	29.47	554.265	70.51	
2	2	1	0	27.94	2.28	642.23	0.4	
3.5	2	3	0	75.40	9.185	407.75	922.23	
3	2	3	0	258.70	0.16	840.24	590.22	
3	3	2	0	119.96	0.16	1341.78	551.915	
2	2	1	0		2.53	909.315	1.285	
3.5	4	3	0	441.37	2.03	318.865	0.4	
2	3	0	0		3.26	614.76	0.4	

3	1	2	0		0.16	1180.825	514.53	
3	4	0	0	157.21	0.16	759.46	303.65	
3.5	4	2	0		0.16	153.705	2373.865	
3	2	3	0		0.16	333.7	566.17	
3	2	2	0		0.16	584.885	618.055	
3	2	2	0	136.48	6.46	492.91	4.095	
3.5	1	3	0		0.16	999.185	703.34	
3	3	2	0		90.365	504.49	824.515	
2	1	0	0	5.51	4.89	293.82	143.81	14.69
2	3	0	0	35.86	5.85	305.98	263.90	18.15
1	1	0	0	7.07	11.60	477.28	174.28	5.19
2	3	0	0	206.61	4.02	284.26	301.30	9.20
2	3	0	0	59.49	2.67	461.43	1070.895	5.724913
1	1	0	0	23.87	5.94	1440.85		2.9246448
1	2	0	0	11.15	8.735	1624.03	746.325	
1	1 or 2	0	0	30.76	9.6	711.865	182.18	
1	1	0	0	29.05	1.29	1452.56	561.11	
1	1	0	0	4.35	1.28	1087.285	513.755	0.8628346
1	1	0	0	5.65	3.135	398.235	26.705	
1	2	0	0	23.50	1.64	403.415	0.4	
2	2	0	0	20.90	13.025	1596.255	58.63	
1.5	2	0	0	22.63	0.16	705.505	663.495	
1	1	0	0	9.92	0.16	746.965	277.75	
1	2	0	0		11.5	432.58	717.305	
3.5	2	3	0	141.30	62.485	842.36	277.75	
	1		0		80.91	507.59	1954.68	
3	3	2	0		8.575	1844.435	604.07	
3	4	1	0		8.415	294.895	1347.02	

3 32

	TGF-baseline
CFRT	5.8444
CHRT	5.24
SBRT	5.724913

pre_il7	pre_il8	pre_il10	pre_il15	2w_il2	2w_ip10	2w_scd40l
22.31	13.54	566.69	23.17	58.92	76.00	1814.00
20.02	11.31	423.23	2.27	21.59	218.00	871.00
2.00	8.01	76.11	1.02	15.82	166.00	379.00
75.89	22.30	284.87	50.35	16.25	753.00	311.00
41.83	6.82	154.31	46.11	12.87	227.00	232.00
13.24	12.49	56.53	17.57	22.41	571.00	234.00
79.36	7.44	227.21	45.28	10.66	1343.00	200.00
2.00	59.38	10.00	7.76	13.83	1174.05	484.28
2.00	24.94	7.85	3.92	2.00	552.40	575.69
2.00	24.24	2.48	2.00	2.91	474.09	1477.81
11.63	19.13	11.67	8.36	5.10	1501.89	572.19
3.20	2.47	7.03	6.77	4.30	774.31	346.77
1.78	12.405	0.39	0.49	0.12	3.2	
1.78	10.685	0.39	2.19	312.2343	406.7901	0
1.78	30.505	95.47	2.24	0.345	3.2	
5.895	47.455	11.57	8.745	0.64	458.245	358.12
6.83	38.41	11.72	11.25	8.06	941.88	429.8
3.2	8.915	9.28	8.895	8.31	559.235	145.105
2.00	13.78	5.37	3.22	0.72	1195.00	786.00
2.00	2.00	38.52	52.82	21.29	153.00	197.00
53.09	22.08	123.47	31.42	11.30	163.00	94.00
2.00	2.00	13.07	5.68	8.18	268.00	61.00
109.66	5.41	1019.70	339.63	80.43	408.00	4003.00
65.00	150.55	1058.56	26.91	178.01	903.00	2428.00
28.16	7.72	525.11	2.74	22.65	301.00	504.00
15.93	29.12	216.01	6.79	41.39	113.00	738.00
24.76	2.00	89.00	21.83	9.38	564.00	145.00
3.20	24.79	6.67	9.18	6.31	382.58	669.37
37.38	2.00	107.73	30.23	5.71	171.00	1841.00
50.31	9.64	150.02	21.36	17.16	153.00	394.00
10.35	3.78	61.68	24.72	9.17	201.00	100.00
75.82	17.66	296.88	54.94	23.23	162.00	445.00
19.11	6.58	378.42	2.00	56.14	380.00	735.00
2.00	9.31	2.00	2.00	2.00	190.00	1070.00
2.00	40.01	27.96	11.74	2.00	335.00	943.00
18.13	17.78	335.44	1.18	95.18	65.00	1678.00
4.74	5.25	27.00	1.96	2.00	173.00	659.00
50.45	16.82	6.23	12.10	15.29	667.39	357.25
2.00	23.56	3.01	3.16	4.95	120.69	195.21
3.20	3.30	7.97	5.76	5.45	477.57	177.94

2.00	33.53	6.94	93.82	113.02	1884.63	156.04
2.00	5.33	17.68	1.89	2.28	449.61	174.20
2.00	46.65	3.89	3.33	0.74	150.64	92.40
2.00	23.33	3.72	1.75	1.52	112.56	123.87
2.00	7.89	19.02	2.79	1.09	222.06	197.84
2.00	9.53	5.09	3.60	9.23	460.45	157.05
2.00	7.44	3.72	1.61	1.68	87.82	158.17
43.86	33.81	7.42	5.80	3.98	741.75	261.21
2.00	5.35	27.60	6.18	4.38	167.98	168.91
2.00	61.13	3.64	9.11	13.66	182.64	257.20
2.00	10.29	6.89	2.21	1.06	380.56	94.75
2.00	18.74	7.74	2.90	2.00	349.64	567.04
2.00	9.58	27.50	4.87	13.09	610.76	477.64
6.96	11.42	46.11	32.83	33.39	474.12	633.80
2.00	38.98	5.29	17.96	136.58	332.74	457.12
13.69	59.73	12.64	75.68	241.71	1848.26	975.98
2.00	6.42	3.48	4.22	2.00	1250.46	340.81
2.00	15.60	6.03	6.41	2.00	434.42	146.96
111.35	47.91	5.61	4.02	2.00	2072.74	265.82
2.00	52.48	5.82	4.39	2.00	524.93	760.11
31.23	7.59	24.63	4.02	2.00	187.61	418.52
52.89	32.49	155.08	91.25	485.53	2112.32	8420.73
2.00	8.39	0.50	6.20	8.94	171.17	1038.56
20.13	29.54	7.09	2.67	12.36	488.88	935.84
2.00	7.46	1.02	2.00	0.45	546.15	363.14
2.00	29.52	12.42	31.63	21.91	546.31	795.22
2.00	12.23	3.48	18.89	2.00	508.76	505.24
3.20	6.31	5.41	5.97	5.65	939.76	352.26
3.20	1.72	5.73	6.39	3.77	312.90	65.09
3.20	0.34	5.65	5.43	4.42	101.54	148.64
3.20	0.34	22.94	6.20	5.73	1080.02	215.39
3.20	14.40	9.00	6.22			
1.78	13.715	6.14	0.49	312.15477	393.2642	0
1.78	18.27	0.39	0.49	0.62	3.2	
16.84	12.815	29.425	18.455	0.12	3.2	
20.36215516	0	166.697098	0	0.12	3.2	
1.78	7.575	0.39	0.49	1.5	3.2	
1.78	32.145	0.39	0.49	0.12	3.2	
1.78	2.875	15.355	1.54	0.955	3.2	
37.905	20.935	103.35	36.875	0.12	15.97	
1.78	12.08	0.39	15.085	2.01	3.2	
4.595	21.015	0.39	4.22	5.8	3.2	

0.64	13.22	6.01	10.855	27.83	921.46	343.13
4.09	4.62	3.405	6.59	0.7	675.075	498.19
0.64	25.445	0.64	0.11	0.64	284.035	346.08
0.64	24.605	4.51	3.765	3.885	411.685	564.52
0.64	10.195	0.71	0.11	0.64	670.255	356.79
0.64	17.715	17.64	0.11	9.955	389.365	500.895
0.64	14.095	3.9	35.435	15.815	375.335	441.285
0.64	8.59	5.76	0.11	5.96	361.48	472.695
0.64	6.615	9.5	0.11	0.64	500.35	374.075
3.2	21.84	12.88	12.16	13.06	316.775	311.03
0.64	17.75	8.865	0.11	3.615	712.87	326.2
0.00	12.96	20.07	2.88	5.79	1072.02	
31.40	20.16	17.75	20.62	4.94	147.31	
0.00	18.47	3.51	2.24	1.20	1944.55	
1.56	9.54	17.87	12.61	4.57	839.06	
0.00	31.71	5.71	5.54	0.00	566.86	
0.00	18.97	0.00	1.10	0.00	1598.11	
17.74	32.48	5.67	11.96	0.00	890.30	
0.00	34.43	9.08	9.24	0.88	826.23	
30.76	25.06	2.16	10.87	1.20	859.80	
8.49	11.65	5.63	4.46	7.80	898.53	
0.00	19.88	0.00	0.00	0.00	2025.06	
2.39	12.44	30.81	7.71	1.915	155.71	1227.52
17.39	12.495	35.275	12.325	2.925	1239.755	443.705
1.45	2.265	6.58	10.41	3.31	678.19	504.605
0.48	31.42	0.215	2.42	0.495	586.24	439.085
80.01	307.485	193.71	98.48	202.87	1115.695	2834.6
3.2	14.15	19.655	8.165	8.31	637.515	780.365
24.555	9.975	47.155	15.305	3.51	685.265	2529.92
0.48	14.02	1.455	0.78	1.18	191.88	2071.94
0.48	12.995	0.905	1.275	3.12	222.27	1171.055
0.46	14.15	8.72	6.615	4.09	200.39	8.22
1.52	9.59	6.37	6.94	1.77	238.97	0.4
0.99	8.42	7.925	8.065	4.09	163.28	0.4
4.11	44.08	7.705	5.31	7.385	465.6	31.35
0.46	11.775	6.48	5.07	2.78	885.765	0.4
3.2	21.46	54.95	0.17	0.16	231.785	357.105
3.2	8.16	19.87	0.17	0.16	858.795	360.795
3.2	9.995	3.33	3.01	0.16	901.29	508.395
0.46	10.32	7.2	5.925	2.715	332.865	0.4
0.46	54.115	14.53	9.99	2.405	3762.15	0.4
0.46	50.735	9.74	5.56	3.02	619.09	0.4

3.2	3.425	0.01	0.17	0.16	665.24	581.965
3.2	4.76	0.01	0.17	0.16	429.025	239.885
9.65	4.795	0.45	6.34	0.16	305.175	1123.735
3.2	11.91	0.01	0.17	0.16	321.775	622.17
3.2	29.39	3.9	3.375	0.16	415.915	779.67
27.41	23.285	26.145	16.91	3.5	480.305	13.805
3.2	5.915	0.01	0.17			
10.04	11.25	27.515	24.08	14.11	110.84	315.31
3.20	7.87	4.81	6.66	4.79	230.97	149.87
3.20	6.83	6.47	6.77	6.40	226.60	162.50
3.20	1.82	4.90	7.64	10.66	447.64	152.85
3.20	0.34	5.59	6.13	4.30	214.36	300.09
0.64	9.55	17.635	0.11	3.96	489.25	1251.215
0.00	29.81	7.13	1.46	6.58	247.16	
8.695	16.25	16.635	12.155	8.365	918.26	601.575
3.2	24.84	11.165	9.56	8.925	646.05	151.15
0.48	13.445	12.97	4.815	1.5	1903.7	398.27
5.305	12.995	18.955	6.18	1.5	1148.245	677.105
2.445	18.77	6.6	8.42	3.32	688.795	29.27
0.46	20.49	4.08	5.64	1.64	404.085	0.4
4.655	26.165	62.7	6.94	7.005	865.055	47.705
3.2	5.52	0.01	0.17	0.16	645.825	972.795
3.2	7.75	0.01	2.94	0.16	786.24	304.31
3.2	28.045	15.065	10.74	8.71	325.37	392.385
3.2	8.4	0.01	9.605	77.79	309.15	568.84
478.045	19.97	4.885	24.47	74.87	1206.33	515.29
17.41	9.455	11.49	9.315	8.365	3266.18	250.68
3.2	63.765	10.29	8.375	8.42	214.96	483.84

TGF-2-week
6.74
3.88
7.46724

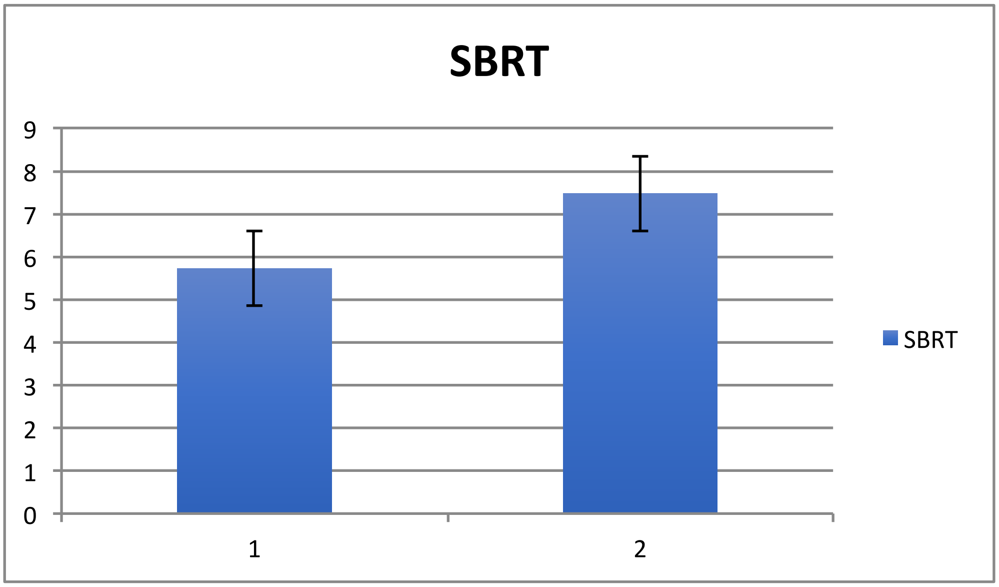

2w_TGFbeta1	2w_il10	2w_il15	2w_il7	2w_il8	4w_il2	4w_ip10
12.65	478.52	30.84	23.21	14.00	51.47	84.28
6.36	165.12	6.79	9.02	11.00	34.59	184.25
8.74	103.89	2.00	4.10	6.00	8.98	231.46
18.50	269.78	49.52	68.12	25.00	17.46	955.05
28.40	217.75	53.88	49.24	7.00	12.56	220.39
17.68	313.89	60.24	86.85	16.00	15.83	471.90
6.74	127.78	39.61	51.47	8.00	11.14	2068.89
2.40	9.36	8.87	2.00	65.73	1.65	1497.18
2.80	19.54	5.03	2.00	18.51	2.00	762.75
19.26	8.71	2.77	2.00	27.29	2.43	2587.33
11.94	10.79	11.03	8.36	13.25	8.58	3655.24
2.18	7.86	7.53	3.20	4.06	4.28	1865.66
3.227998	0.39	0.49	1.78	8.59	1.49	3.2
	166.697098	103.828487	20.773701	0	0.485	3.2
4.9499275	21.115	14.565	7.07	59.345	0.12	3.2
0.5600942	1.705	2.06	0.64	34.545	0.67	375.2
	3.115	11.535	0.48	18.95	9.22	1342.145
	9.47	8.895	3.2	4.755		
10.66	54.64	3.54	4.04	21.00	12.00	125.24
17.03	31.48	26.62	6.71	2.00	35.70	304.91
2.75	18.13	12.05	2.00	2.00	10.45	396.22
5.06	57.16	17.47	16.23	2.00	8.02	293.24
21.20	542.26	219.41	68.59	3.00	25.67	506.78
29.77	1148.67	29.82	68.64	130.00	138.24	1379.90
7.99	181.82	2.11	7.14	5.00	2.00	366.19
13.10	309.77	15.41	19.78	19.00	25.79	465.63
10.38	83.11	20.39	21.41	2.00	7.85	851.16
6.73	5.88	8.42	3.20	11.18		
14.39	78.88	4.90	10.80	2.00	16.05	140.86
17.02	138.03	43.74	42.85	2.00	8.57	318.85
3.30	93.24	20.99	11.80	2.00	13.65	217.36
40.00	371.05	64.83	93.32	15.00	14.37	181.30
10.22	425.07	4.03	20.43	10.00	67.76	379.99
3.59	22.06	3.55	2.00	5.00	2.00	317.23
2.65	2.75	13.40	2.00	30.00	2.00	273.20
19.21	681.48	13.57	39.23	9.00	23.85	148.66
2.33	161.41	7.77	11.91	5.00	2.00	292.62
1.09	4.49	13.04	43.64	17.73	2.49	527.45
14.96	3.43	5.35	2.00	44.31	2.24	297.22
1.34	7.08	5.93	3.20	2.95		

1.40	6.46	72.46	2.00	16.68	308.38	4862.03
2.40	24.90	2.40	2.00	7.14	3.16	881.70
1.28	18.83	5.51	2.00	31.32	0.41	417.15
2.80	2.97	2.50	2.00	23.60	0.42	172.16
10.84	9.55	3.34	2.00	9.17	0.04	397.35
1.60	7.12	5.67	2.00	11.50	6.97	1018.03
4.00	5.18	2.07	2.00	9.85	4.65	177.37
2.44	11.47	7.11	3.20	33.15	4.32	737.29
1.40	10.12	2.89	2.00	5.01	11.21	136.89
4.19	3.47	6.05	2.00	61.98	13.17	283.40
3.88	7.38	2.47	2.00	5.77	0.13	170.01
2.40	50.35	3.45	2.00	14.36	2.00	320.26
3.20	23.80	9.50	2.00	12.42	2.00	605.86
1.28	18.04	23.08	2.00	7.78	2.00	260.73
3.87	4.56	20.64	2.00	35.91	21.18	567.80
1.00	17.70	70.77	18.14	55.77	315.99	1831.26
2.48	3.84	4.97	2.00	8.49	2.00	2140.98
1.60	11.14	9.70	2.00	6.29	2.00	1514.90
1.60	14.39	6.02	26.63	33.97	2.00	802.75
1.92	4.15	4.18	2.00	53.89	2.00	869.29
2.52	16.70	5.13	12.59	4.22	2.00	337.57
4.86	94.48	266.82	7.25	17.40	472.90	1720.46
14.01	4.74	8.41	2.00	7.83	2.25	567.86
18.20	9.85	4.64	2.00	26.79	37.30	577.17
10.56	1.42	4.16	2.00	5.65	1.04	313.68
12.01	5.61	16.96	2.00	16.99	12.90	414.88
1.15	1.42	4.60	2.00	12.87	2.00	525.74
2.85	6.40	7.53	3.20	10.59	7.52	775.64
1.10	4.93	6.96	3.20	2.38	3.96	1040.52
3.38	8.67	5.55	3.20	0.34	4.00	771.70
5.21	43.99	8.27	3.20	4.87	4.50	797.70
0.00					4.10	225.18
	0	102.869466	0	0	312.1749	413.5169
6.397283	1.085	1.505	1.78	8.82	1.63	3.2
7.1173095	47.02	18.29	17.83	6.735	0.2	3.2
4.536606	0.625	2.24	1.78	75.8	0.12	3.2
4.8535345	0.39	4.875	1.78	5.33	0.345	3.2
4.542448	0.39	0.49	1.78	9.595	1.61	3.2
7.767232	57.33	12.25	2.565	39.21	0.12	3.2
4.5994075	74.41	27.985	29.21	15.47	2.61	17.705
3.914433	1.755	0.49	11.485	9.92	0.425	3.2
9.2058245	1.435	17.465	11.145	19.265	2.85	3.2

1.3884734	5.49	25.68	9.8	14.16	12.085	549.295
4.1764087	0.96	0.11	0.64	3.99	4.275	721.74
2.4584632	0.64	0.11	0.64	16.53	55.055	557.135
7.419325	7.59	12.01	0.64	18.025	3.725	508.225
1.4355404	0.64	7.145	0.64	10.195	2.355	844.965
4.1167905	0.64	0.11	0.64	12.67	12.97	877.555
12.5104055	4.045	46.41	9.31	16.535	18.335	511.455
5.2495363	5.89	0.11	0.64	11.31	4.275	334.755
1.7916807	11.085	0.11	0.64	8.815	0.67	489.78
	11.61	13.15	3.2	23.71	11.105	166.315
1.7681472	7.165	6.52	0.64	30.03	4.7	726.57
3.085288	3.85	12.45	0.00	20.73	5.60	981.87
7.1864144	9.08	9.11	0.00	15.34	5.09	175.51
3.2577432	2.21	2.07	0.00	8.15	0.00	2929.80
3.5884792	3.20	9.84	0.00	4.38	0.00	758.37
1.3229376	11.45	9.86	0.00	45.35	4.87	418.38
2.5324864	0.00	0.87	0.00	19.36	1.34	1362.21
5.0602544	5.97	14.64	0.00	26.74	2.06	1331.51
1.6182376	13.33	12.81	0.00	29.65	1.86	549.33
2.3340448	42.23	10.64	37.56	19.95	0.00	1282.97
9.9693216	11.76	11.49	8.49	17.30	4.77	1122.78
4.8618128	0.00	0.00	0.00	17.25	0	1430.282
3.0876738	12.275	8.55	0.48	9.785	1.07	276.415
5.5892062	36.255	15.455	19.815	15.025	4.85	853.335
1.2806978	4.5	10.54	0.48	3.23	1.29	498.98
3.7116452	0.215	4.27	0.48	33.36	0.39	1899.26
5.2447514	200.035	107.085	92.06	360.285	171.98	1517.08
	11.05	8.145	3.2	17.97	8.63	2585.795
9.7113702	39.33	16.78	24.685	22.41	13.65	862.815
2.870272	2.01	2.94	0.48	19.23	1.5	261.685
2.474996	6.165	5.495	0.48	18.715	2.425	369.26
	6.925	5.395	0.46	9.21	4.78	292.315
	5.765	7.22	0.46	8.065	1.9	216.575
	7.93	7.425	0.46	6.2	2.78	217.115
	6.04	5.76	2.73	24.88	2.9	208.34
	9.74	7.425	0.46	18.15	2.95	5489.615
	48.05	4.61	3.2	9.915	0.16	688.33
	0.01	7.125	3.2	30.93	0.16	1393.33
	5.37	4.61	3.2	12.595	0.16	1730.34
	7.255	8.39	2.155	12.79	2.025	291.375
	51.535	15.245	0.46	85.59	4.555	3864.47
	6.425	8.79	0.46	43.545	2.96	999.08

	0.01	0.17	3.2	6.12	0.16	424.665
	0.01	0.17	3.2	2.52	0.16	1388.635
	0.24	12.72	12.91	7.29	0.16	423.935
	0.01	1.535	3.2	7.255	0.16	136.715
	6.04	12.715	3.2	13.725	0.16	484.855
	20.7	19.76	21.925	19.74	1.9	977.27
					8.39	581.985
	16.015	12.54	3.2	1.975	11.885	74.595
13.08	4.67	7.00	3.20	4.92	5.79	902.84
15.59	7.03	7.15	3.20	1.08	5.55	585.65
8.38	5.01	8.35	3.20	2.01	13.88	853.13
7.47	5.64	6.34	3.20	0.34	4.44	179.01
4.7349371	14.47	0.11	0.64	9.08	1.84	341.24
2.1025296	20.56	6.05	0.00	8.94	0	1463.811
	15.495	10.95	7.855	12.13	10.395	1654.735
	10.13	8.9	3.2	8.54	8.205	388.96
	12.97	5.355	0.48	17.62	1.5	1839.6
2.5935788	22.72	8.27	8.77	17.095	1.385	1505.73
	6.59	9.11	1.41	17.495	9.975	667.505
	4.505	5.97	0.46	13.755	1.9	970.585
	35.155	6.125	3.265	17.59	0.16	851.63
	0.01	0.17	3.2	4.7		
	0.01	2.425	3.2	13.315	0.16	745.335
	18.93	11.39	3.2	23.905	9.22	378.13
	27.19	26.325	40.91	13.61	52.03	247.17
	12.05	26.19	195.445	54.92	30.76	926.83
	10.45	9.67	39.22	5.205	8.74	8006.825
	11.325	9.625	3.2	55.215	8.52	352.13

4w_scd40l	4w_TGFbeta1	4w_il10	4w_il15
1180.83	9.91	381.16	25.56
1086.06	11.47	302.45	3.89
267.54	6.94	93.94	2.00
288.09	17.56	292.41	50.58
210.51	25.30	172.33	51.76
184.67	17.14	186.88	40.06
310.74	8.76	181.72	47.87
328.96	2.48	10.83	9.07
587.78	2.20	8.28	5.05
835.26	1.49	0.89	2.00
586.67	2.93	12.15	9.70
346.74	0.37	7.96	7.32
	6.607595	0.39	0.49
	6.686462	0.39	2.52
	13.5230625	23.605	3.875
242.69	0.9256479	0.64	0.11
800.68	1.3371658	6.16	10.83
590.00	6.44	132.55	16.08
320.61	9.06	144.01	53.64
127.47	2.29	11.50	11.68
86.94	5.18	47.49	17.83
514.94	7.69	129.75	67.78
1830.01	26.09	1070.54	33.43
447.56	2.84	2.00	7.60
494.22	24.01	238.95	28.45
701.42	17.22	20.35	14.04
	0.05		
187.61	8.16	72.70	35.70
371.13	7.63	83.12	22.92
249.03	10.64	186.50	43.74
439.52	10.39	130.10	35.82
813.63	12.38	569.43	12.07
1128.04	2.54	2.00	4.51
919.38	2.46	10.03	12.56
772.77	7.73	280.43	9.08
871.37	1.84	15.55	9.58
265.62	4.77	7.16	6.81
322.50	13.25	4.96	4.68
	3.26		

359.12	1.32	20.84	131.10
158.37	9.57	51.46	2.70
134.80	1.20	3.81	3.06
218.77	3.20	3.38	3.03
192.57	8.26	8.01	4.16
225.30	3.60	7.12	5.71
359.82	4.40	5.44	2.03
138.99	3.01	11.73	8.48
301.88	1.20	33.91	4.11
281.78	3.81	4.75	8.30
206.35	9.81	5.44	2.73
476.39	2.40	6.35	3.29
385.20	1.28	20.94	4.88
401.47	2.48	8.07	8.14
327.78	1.40	3.64	8.54
1226.61	1.00	27.27	95.97
349.46	1.20	4.46	5.24
334.02	2.52	10.25	9.62
557.51	3.04	3.74	3.84
705.29	1.92	6.40	4.22
320.95	2.00	10.48	5.33
10000.00	3.01	35.53	124.30
629.06	12.27	6.22	5.72
1297.28	9.53	1.81	6.20
267.82	11.94	1.41	4.65
512.88	3.06	9.04	23.01
1620.91	3.88	4.47	3.17
268.77	5.29	7.81	8.16
137.48	-0.03	7.84	8.33
116.12	1.95	10.78	6.30
120.05	2.10	57.49	7.96
34.53	4.14	52.41	11.40
0		167.550968	103.05331
	8.5719675	2.45	4.09
	5.54143	27.505	19.385
	4.367188	0.39	0.49
	4.2342825	2.78	4.98
	6.137314	15.05	14.19
	7.3013325	64.62	13.905
	11.447692	65.105	23.74
	3.9275775	1.09	12.075
	9.8046295	1.31	9.195

354.135	1.8701257	1.28	11.76
464.695	10.400235	3.68	1.565
302.005	1.457505	9.14	31.15
692.99	4.1011015	6.95	19.455
194.11	0.8503407	3.075	8.95
842.275	4.9106539	12.44	0.11
362.77	8.9270379	16.43	46.285
418.65	1.7116668	8.055	1.565
426.62	2.7534164	14.66	0.11
287.875		11.9	12.275
278.755	1.6567553	7.395	6.845
	4.479104	5.22	14.75
	3.1278112	2.88	6.32
	1.7056464	9.89	4.46
	0.9614904	14.74	5.74
	2.8419608	3.85	13.96
	4.9941072	0.00	5.28
	13.1113136	8.32	20.41
	3.92394	8.80	20.10
	1.254428	0.00	13.62
	2.6553312	15.63	10.27
	6.4446208	0	0
599.8	0.724488	15.895	8.555
536.55	4.4400824	22.3	11.035
492.79		0.905	14.14
401.91	0.9757706	4.775	7.71
1752.8	0.5748478	241.66	122.3
224.335		12.8	8.745
1538.285	6.5293984	25.645	15.38
426.24		2.56	3.47
1474.515	1.825614	5.33	5.08
22.95		7.59	6.695
0.4		96.2	9.99
3.69		9.51	5.31
0.4		8.72	7.99
3.44		15.645	9.87
969.29		44.23	6.84
433.275		0.01	4.9
384.17		8.13	5.19
0.4		6.81	8.55
22.79		24.135	16.59
0.4		13.305	11.425

1066.695		0.01	1.24
350.985		0.01	0.48
868.235		0.01	12.72
425.715		0.89	5.12
951.965		4.195	9.33
782.1		24.17	16.43
350.445		8.475	10.925
283.46		13.775	12.445
469.40	7.72	7.27	4.97
162.50	5.05	5.57	6.59
371.47	4.77	4.95	8.83
210.88	2.61	6.11	6.77
1230.635	6.5062252	10.725	0.11
		0	0
572.58		19.11	13.03
149.12		12.755	10.19
342.48	0.9305962	10.745	6.82
731.525	5.3153364	20.91	8.695
101.07		9.68	15.44
0.4		5.82	6.33
705.12		9.22	0.78
664.005		0.01	1.54
645.89		15.5	11.115
1008.085		36.675	22.61
1869.38		18.035	18.055
502.43		12.41	11.4
328.18		14.51	10.1
